# Mycoplasma contamination of leukemic cell lines alters protein expression determined by reverse phase protein arrays

**DOI:** 10.1007/s10616-018-0244-2

**Published:** 2018-09-06

**Authors:** Fieke W. Hoff, Chenyue W. Hu, Amina A. Qutub, Yihua Qiu, Elizabeth Graver, Giang Hoang, Manasi Chauhan, Eveline S. J. M. de Bont, Steven M. Kornblau

**Affiliations:** 1 Department of Pediatric Oncology/Hematology, Beatrix Children’s Hospital, University Medical Center Groningen, University of Groningen, Groningen, The Netherlands; 2 Department of Bioengineering, Rice University, Houston, TX USA; 3 Department of Leukemia, The University of Texas MD Anderson Cancer Center, 1515 Holcombe Blvd, Box 448, Houston, TX 77030-4009 USA

**Keywords:** Mycoplasma, RPPA, Cell lines, Proteomics

## Abstract

Mycoplasma contamination is a major problem in cell culturing, potentially altering the results of cell line-based experiments in largely uncharacterized ways. To define the consequences of mycoplasma infection at the level of protein expression we utilized the reverse phase protein array technology to analyze the expression of 235 proteins in mycoplasma infected, uninfected post treatment, and never-infected leukemic cell lines. Overall, protein profiles of cultured cells remained relatively stable after mycoplasma infection. However, paired comparisons for individual proteins identified that 18.7% of the proteins significantly changed between the infected and the never-infected cell line samples, and that 14.0% of the proteins significantly altered between the infected and the post treatment samples. Six percent of the proteins were affected in the post treatment samples compared to the never-infected samples, and 7.2% compared to treated cells that had never had mycoplasma infection before. Proteins that were significantly altered in the infected cells were enriched for apoptotic signaling processes and auto-phosphorylation, suggesting an increased cellular stress and a decreased growth rate. In conclusion, this study shows that mycoplasma infection of leukemic cell lines alters the proteins expression levels, potentially confounding experimental results. This reinforces the need for regular testing of mycoplasma.

**Electronic supplementary material:**

The online version of this article (10.1007/s10616-018-0244-2) contains supplementary material, which is available to authorized users.

## Introduction

Mycoplasma contamination of cell lines is one of the major problems of cell culturing, with infection present in 5–30% of the cell lines, despite significant improvements in diagnostic and therapeutic possibilities to detect and eliminate mycoplasma contamination (Drexler and Uphoff 2002; Drexler et al. 2017; Uphoff and Drexler 2002). Because mycoplasmas lack a cell wall, their flexibility enables them to pass through anti-bacteriological filters which facilitates the contamination of media and cell cultures. The lack of a cell wall also allows direct and tight contact with the host cytoplasmic membrane that may result in cell fusion (Drexler and Uphoff 2002; Nikfarjam and Farzaneh 2012; Rottem and Barile 1993). Mycoplasma infected cell cultures frequently cause further spreading of contamination by the ease of droplet formation during sample handling (Drexler and Uphoff 2002). In contrast to bacteria and fungi, mycoplasmas grow relatively slowly and do not cause acute cellular damage. They are resistant to most of the commonly used antibiotics (Drexler and Uphoff 2002; Nikfarjam and Farzaneh 2012). Therefore, mycoplasmas can live on the surface of eukaryotic host cells for an extended period of time (Miller et al. 2003; Zhang et al. 2000). Moreover, the mycoplasmas genome lacks the ability to provide for their own metabolism or replication which makes them dependent on their host cells for survival. This dependency leads to competition for nutrients (e.g. arginine which is needed for protein synthesis of the host), which can disrupt cell integrity and in turn alter the host cell function (Miller et al. 2003; Rottem and Barile 1993). Additionally, as histone modification is reliant on arginine, arginine depletion is thought to disrupt histone synthesis and so influences gene regulation (Rottem and Barile 1993; Rottem and Naot 1998).

As leukemic cell line experiments are an essential tool to gain biological knowledge, it is important to know the consequences of mycoplasma contamination and to establish stable and reproducible cell cultures. A study by Miller et al. (2003) already showed that mycoplasma infection alters gene expression of hundreds of genes in cultured human cells, and Gedye et al. (2016) reported poorly reproducible results from an aliquot of a cell line that was later found to be infected with *Mycoplasma hyorhinis*. However, limited knowledge is available on the effects of mycoplasma on protein expression. In this study we determined protein expression levels of 235 proteins in mycoplasma infected and non-infected cell lines along with post treatment mycoplasma-free versions using the reverse phase protein array (RPPA) technology.

## Materials and methods

### Leukemic cell culture

Leukemic cell line samples (n = 52) derived from either pediatric or adult AML (e.g. Kasumi-1, HL-60, OCIAML3), ALL (e.g. Jurkat, REH), CML (e.g. KBM5) and lymphoma (e.g. Raji) including various molecular modifications were obtained from different laboratories. Cell lines underwent Short Tandem Repeat DNA fingerprinting for cell line identity confirmation and were cultured according to the ATCC cell culture methods. All cell lines were tested for mycoplasma using the mycoplasma Polymerase Chain Reaction (PCR) detection kit (Applied Biological Materials Inc., Richmond, BC, Canada, Catalog No. G238). Of these, 16 were positive for mycoplasma infection and from these a sample was collected for protein isolation for the ‘‘infected’’ samples. Infected cell lines were then treated with 25 µg/mL Plasmocin™ Treatment (InvivoGen, San Diego, CA, USA, Catalog# ant-mpt, version# 15B23-MM) for 2 weeks, and PCR was repeated immediately post treatment to check their mycoplasma status. Cell cultures that were successfully treated (all except one), were then cultured for another 2–4 weeks and again tested by PCR to verify if cell lines remained negative during culturing. From each cell line culture cells were collected for the production of protein for the “post treatment” sample from this ~ 4-week sample. As it is conceivable that mycoplasma infection could have permanent effects on cell line protein expression patterns, we attempted to assess this by obtaining another sample of the same cell lines, that had never had a mycoplasma infection, as best as we could ascertain, for comparison. Those cell lines were cultured similarly and checked by PCR on mycoplasma before preparation of protein sample from the ‘‘never-infected’’ sample. For three infected cell lines, no never-infected counterpart could be found. In addition, to assess the effect of Plasmocin™ treatment on protein expression, four never-infected cell lines were treated and added to the array. In summary, a total of 52 samples were available for comparison of protein expression between the different mycoplasma states; twelve cell lines had a complete set of an infected, an uninfected post treatment and a never-infected sample, one cell line had an infected and never-infected sample, three cell lines had an infected and post treatment sample, and four cell lines that had a never-infected and post treatment sample that had never been infected before. More information regarding the cell lines, including type, mycoplasma status, disease and culture method are provided in Supplemental Table S1.

### Reverse phase protein array methodology

RPPA was used to determine the protein expression of 52 leukemic cell line samples and ten normal CD34+ bone marrow samples. Methods and antibody validation techniques are fully described in previous publications (Kornblau et al. 2009, 2010; Tibes et al. 2006). Briefly, whole cell lysate protein preparations were prepared and normalized to a concentration of 1 × 10^4^ cell/µL and printed in five serial (1:2) dilutions onto slides along with normalization and expression controls. Slides were probed with 235 strictly validated antibodies and a secondary antibody to amplify the signal (Hunyady et al. 1996), and finally a stable dye was precipitated. A ‘‘Rosetta Stone’’ of manufacturer, antibody name (HUGO and MiMI), and primary and secondary antibody dilution can be found in Supplementary Table S2. The stained slides were analyzed using Microvigene^®^ Software (Vigene Tech, Carlisle, MA, USA) to produce quantified data.

### Data normalization

*SuperCurve* algorithms were used to generate a single value from the five serial dilutions (Hu et al. 2007). Loading controls (Neeley et al. 2009) and topographical normalization (Neeley et al. 2012) procedures were performed to account for protein concentration and background staining variations. Since all samples had replicates, the average expression level of the replicates was used as a single expression level. All protein expression levels were shifted relative to the median of the normal CD34+ bone marrow samples.

### Statistical procedure

The unsupervised hierarchical clustering procedure was performed using the Ward-linkage rule. Principal component analysis was employed to further visualize the distribution of each cell line in relation to their mycoplasma status. Paired Wilcoxon signed-rank test was applied to compare protein expression of individual proteins between the three sample conditions [infected vs. never-infected (n = 13), infected vs. uninfected post treatment (n = 15), never-infected vs. uninfected post treatment (n = 12)]. To compare changes in protein expression between the never-infected versus samples post treatment that were never infected before (n = 4), we applied the Student’s *t* test, because the Wilcoxon signed-rank test could not be used for too small numbers of paired samples. All statistical analyses were performed using R (Version 1.0.136^®^ 2009–2016 RStudio, Inc., Boston, MA, USA).

### Gene ontology enrichment analysis

Protein names were uploaded in the Database for Annotation, Visualization and Integrated Discovery version 6.8 and analyzed for gene ontology of biological processes. Biological processes that were significantly enriched (false-discovery rate corrected q-value < 0.05) were selected.

## Results and discussion

**Fig. 1:**
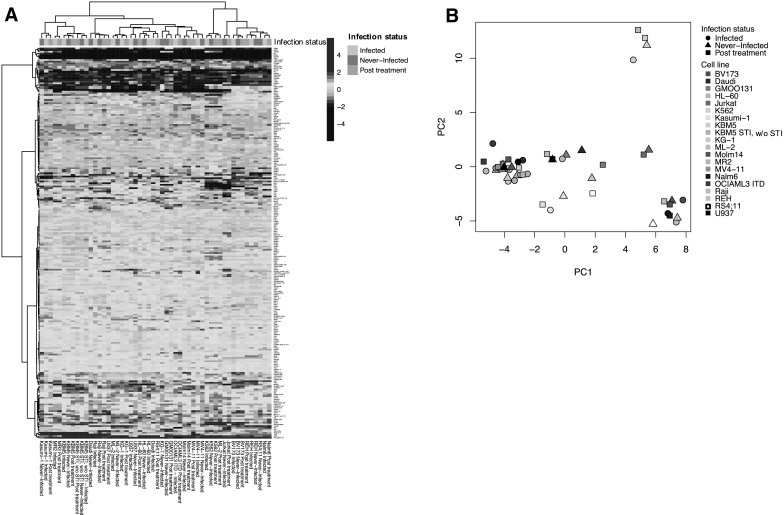
Relative protein expression levels for the cell line samples indicating their mycoplasma status. **a** Heatmap comparing overall protein expression for all cell line samples relative to the normal CD34+ cells. Each row represents an individual protein and each column shows one cell line sample. Annotation bar: infected (green), never-infected (blue), and post treatment (red). **b** Principal component analysis for all 52 samples does not show separation based on mycoplasma status. (Color figure online)

**Fig. 2:**
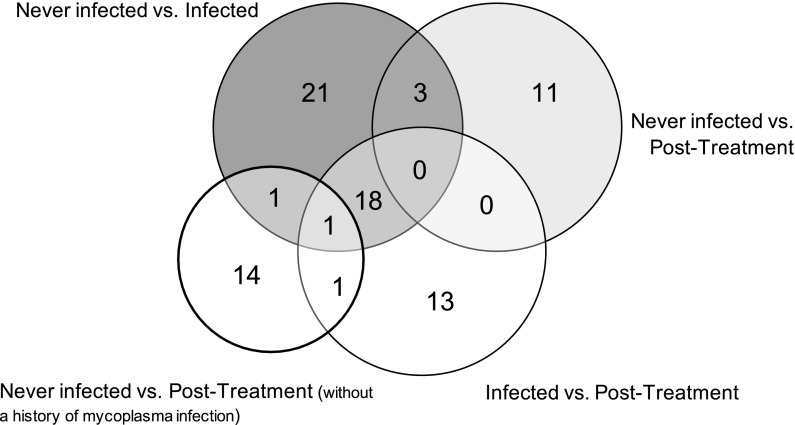
Venn diagram for the number the altered proteins. Overlapping and non-overlapping proteins that were changed in each sample comparison based on the Wilcoxon signed-rank test (alpha = 0.05). Nineteen proteins were affected between the infected and both the post treatment and never-infected cell samples

Protein expression levels for all 235 antibodies were determined and compared between the four subsets of samples; infected, uninfected post treatment, never-infected, and post treatment samples from cells that were never infected before. Globally, based on unbiased hierarchical clustering there was no segregation of the infected samples from the uninfected post treatment or the never-infected samples, as the different samples from a given cell line tended to co-segregate regardless of mycoplasma status (Fig. 1). However, three outliers were observed between the never-infected and infected samples compared to the post treatment samples (e.g. RS4;11, ML-2 and U937), suggesting that antibiotic treatment can affect the protein profiles of the cell lines more than mycoplasma infection in at least some cases. Moreover, when the expression levels of individual proteins were assessed with respect to mycoplasma infection status, several proteins showed changes in their expression as shown in Table 1. Most differences were observed between the infected cells and either the uninfected post treatment state (n = 33/235, 14.0%) or the never-infected state (n = 44/235, 18.7%). Fewer alterations were observed between the never-infected cells and the uninfected post treatment cells (n = 14/235, 6.0%) and between the never-infected cells and the same post treatment cells (n = 17/235, 7.2%). Most of the proteins that exhibited significant alterations in the infected cells had lower protein expression levels in the infected samples compared to the never-infected samples (n = 39/44, 88.6%) and compared to the post treatment samples (n = 32/33, 97.0%). A significant part of the proteins with altered expression in the mycoplasma infected cell lines were similarly affected compared to both the uninfected post treatment samples and the never-infected samples (19 of 44 for infected vs. never-infected and 19 of 33 for infected vs. uninfected post treatment), suggesting a core set of mycoplasma infection altered proteins (Fig. 2). The frequency with which protein levels differed between the various conditions were all above 5%, the expected false positive rate for that *p* value. These observations are in line with our expectations based on the mRNA literature (Miller et al. 2003) that we expected to find differences between the four sample conditions, due to the effect of mycoplasma infection, and we suspected that we would also observe a residual effect of the treatment to eradicate mycoplasma on the cells. All significantly altered proteins together with their median expression are listed in Supplemental Table S3.

**Table 1:** Paired comparisons of protein expression for individual proteins

# Significantly different proteins between the sample conditions		
Comparison	Number of paired samples	Number of affected proteins (%)
Infected versus post treatment	15	33 (14.0)
Infected versus never-infected	13	44 (18.7)
Never-infected versus post treatment	12	14 (6.0)
Never-infected versus post treatment without any mycoplasma history	4	17 (7.2)

Cell lines for which more than one protein sample was collected were used to assess differences in proteins expression due to mycoplasma status

**Fig. 3:**
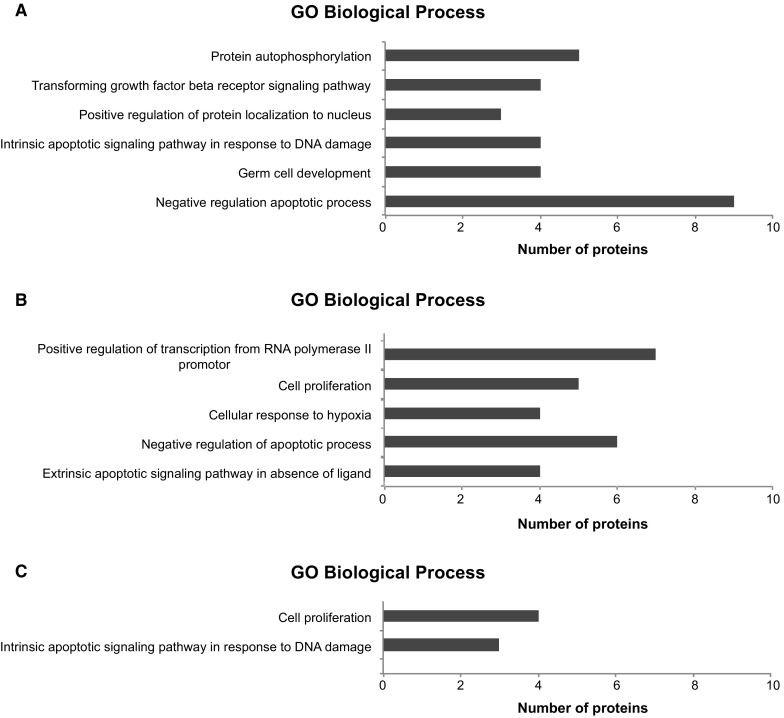
Gene ontology enrichment analysis for the significantly altered proteins. Bar graph showing the significantly enriched Biological Processes for 44 proteins that were altered between the infected and never-infected cell culture samples (**a**), the 33 proteins that were changed between the infected and post treatment samples (**b**), and for the 19 proteins that were altered in both the never-infected and the post treatment samples in comparison to the infected cells (**c**). Biological processes with an false-discovery rate corrected q-value < 0.05 were considered significant

To then globally assess the effect of mycoplasma on the cell biology, pathway analysis was performed for the gene ontology (GO) biological processes (Fig. 3a–c). Proteins that were changed in the infected samples compared to the never-infected samples were primarily involved in the apoptotic processes as well as in protein auto-phosphorylation and germ cell development. Proteins that were changed in infected cell lines compared to the uninfected post treatment cell samples also showed changes in the apoptotic pathway, however GO processes were also significantly enhanced for transcription regulation, cell proliferation and cellular stress. No significant enriched processes were found for the 14 significantly altered proteins from the comparison of never-infected cells and uninfected post treatment samples and for the 17 proteins that changed after treatment of the never-infected cells (likely due to the small number of proteins). The 19 overlapping proteins that were altered between both the uninfected post treatment and the never-infected cell lines compared to the infected cells were enriched for the apoptotic-signaling pathway as well as for cell proliferation.

Taken together, in this study we demonstrated that mycoplasma infection of leukemic cell lines alters the protein expression levels of individual proteins, whereas the overall protein profiles remained relatively stable. Subsequent pathway analysis of the significantly changed proteins in the infected cells compared to the post treatment or the never-infected samples showed significant enrichment for apoptotic signaling processes in particular, but also showed changes in proteins involved in protein auto-phosphorylation and cell proliferation. This may suggest that mycoplasma infection leads to an increased cellular stress response and a reduced cell growth. Also, as infected cells expressed lower protein levels for the majority of the significantly altered proteins compared to their never-infected and uninfected post treatment counterparts, this might imply that the infected cell lacks sufficient energy to synthesize their normal protein levels, as they have to share their metabolism with the mycoplasmas, but that once the mycoplasma is eliminated their metabolism is at least partially restored. However, a larger study should be performed on mycoplasma infected cell cultures to assess the effect on cell biology, as well as to assess the additional effect of Plasmocin™ treatment on the protein expression. In addition, because the majority of the clean counterparts of the infected cell lines originated from different laboratories than the infected and post-treatment samples, and were thus previously cultured under different circumstances, a study that will artificially infect negative cell lines to keep culturing conditions between cell cultures stable, will likely result in more accurate comparisons between cell culture samples.

In order to establish clean cell cultures to make cell line experiments more reliable and reproducible, we strongly encourage the regular testing of cell cultures for mycoplasma and to treat cell lines if cultures test positive to avoid having the consequences of altered expression of these proteins confounding the experimental results. Yet, as few alterations remained between the post treatment samples and the never-infected samples in our cohort, and as changes in protein expression were observed after treatment of never-infected cells, additional cell culturing for more than 2 weeks after completion of the antibiotic treatment, or taking a clean cell line aliquot, should also be considered.

## Electronic supplementary material

Below is the link to the electronic supplementary material.

Supplemental Table S1. Cell line information for the 52 samples included in the analysis. Information is provided regarding mycoplasma status, disease, source, and the ATCC culture method (XLSX 175 kb)

Supplemental Table S2. A “Rosetta Stone” of the antibody and protein nomenclature. This table contains information about the RPPA modified antibody name, the HUGO gene symbol and post-translational modification, and the MiMI and the full protein name. Secondary, RPPA staining details contain information about the manufacturer of each antibody, together with the antibody name, the antibody source, catalog number, and the primary and secondary antibody dilutions (XLSX 35 kb)

Supplemental Table S3. Paired comparisons between the proteins expression levels of the infected, post treatment and non-infected cells. Paired comparison between the protein levels of infected vs. non-infected (n=13) samples, infected vs. post treatment (n=15) samples and post treatment vs. non-infected (n=12) using the Wilcoxon signed-rank test. The median expression is showed for the samples in each comparison (XLSX 52 kb)
